# Autonomous object tracking with vision based control using a 2DOF robotic arm

**DOI:** 10.1038/s41598-025-97930-3

**Published:** 2025-04-18

**Authors:** Umesh Kumar Sahu, Mebin K. S., Abhinav K., Muhammed Muzammil P, Ankur Jaiswal, Umesh Kumar Yadav, Santanu Kumar Dash

**Affiliations:** 1 Department of Mechatronics, Manipal Institute of Technology, Manipal Academy of Higher Education, Manipal, Karnataka 576104 India; 2 Department of Electrical Engineering, Malaviya National Institute of Technology, Jaipur, Rajasthan 302017 India; 3 TIFAC-CORE, Vellore Institute of Technology, Vellore, 632014 India

**Keywords:** Deep learning, Feature based tracking, Object tracking, 2-DOF robotic arm, Image-based visual servoing, Engineering, Mathematics and computing

## Abstract

The tracking of moving object by implementing robot manipulator is one of the challenging task for many applications such as manufacturing, agriculture, logistics, healthcare, space, military, entertainment, etc. In the deployment of robotic manipulators with real-time object tracking for aforementioned important applications, the proper sensor surveillance and ensuring stability are major challenges. The purpose of this study is to design a precise and responsive object-tracking system by eliminating the complexities related to tedious mechanisms, rigidity, requirement of multiple sensors, etc. which are commonly associated with traditional systems. The robotic arms can be effectively designed to track moving objects autonomously with vision-based control. In comparison with different classical and traditional servoing approaches, the image-based visual servoing (IBVS) is more advantageous in vision-based control. The present article describes a new approach for IBVS-based tracking control of 2-degree-of-freedom (DOF) robotic arm by including object identification and trajectory tracking based crucial components. To solve the issues associated with IBVS, an accurate deep learning-based object detection framework is employed. The presented framework is utilized to detect and locate the objects in real-time. Further, an effective vision-based control technique is designed to control the 2-DOF robotic arm with the help of real-time response of object detection system. The validation of proposed control strategy is done by performing a simulation and experimental investigations with CoppeliaSim robot simulator and 2-DOF robotic arm, respectively. The findings reveal that the proposed deep learning controller for the vision-based 2-DOF robotic arm achieves good levels of accuracy and response time while performing visual servoing tasks. Furthermore, thorough discussion on possibility of using data-driven learning technique has been explored to improve the robustness and adaptability of the presented control scheme.

## Introduction

A robotic arm replicates the functions of human arm and can be built to perform a variety of jobs. The arm consists of interconnected links with joints that allow for rotational and linear range of motion, allowing the robot to manipulate the objects with precision and flexibility^1,2^. Robot arms have become increasingly popular across a variety of fields which include industrial automation, agricultural innovation, traction and logistics, biomedical engineering, entertainment, etc.^3–5^. These systems use a variety of sensors, including temperature, radiation, colour, and weight^6^. However, there is a growing trend towards employing single-lens cameras and computer vision techniques to independently conduct jobs with greater precision, potentially replacing the need of several sensors^7,8^. A vision-based object tracking system can enhance the precision and capabilities of robot arms^9^.

A visual-based control or visual servoing (VS) using computer vision enables a robot to view and interact with its environment^10,11^. With this approach, the robot arm can adapt the changes in the environment and perform tasks with greater precision and flexibility, as if it has its own set of eyes to guide its actions. It also avoids conventional method of training a robot, and it has better accuracy and controllability^12,13^. Visual-based robot control relies on feedback from a camera system, which calculates the error between the desired and provided visual information to generate commands for robot’s actuators^14^.

There are two camera configurations such as eye-in-hand (EIH) and eye-to-hand (ETH), depending on where the camera is positioned in the control loop. In EIH configuration, camera is rigidly mounted on the end-effector, and its position is fixed relative to the robot’s pose. Whereas in ETH configuration, camera observes the robot and its surroundings within its workspace. EIH configuration provides a precise view of the scene, but it has limited interaction with the entire workspace whereas ETH configuration provides a global view^15^. In addition, visual servoing have three methods focused on geometric features, Image-Based visual servoing (IBVS)^16,17^, Position-Based visual servoing (PBVS)^18^ and hydrid visual servoing (HVS)^19^. PBVS is a technique that involves establishing a relationship between the robot’s position and attitude, and the image signal using camera parameters. This approach offers the advantage of obtaining more mature control methods and separating visual processing from robot control. However, there are challenges, such as the accuracy of the position and attitude information extracted from the image and the possibility that the robot or reference object may not always be within the camera’s field-of-view^18^.

On the other side, IBVS involves comparing the real-time measured image feature with a reference feature and with the resulting feature error for feedback to form closed-loop feedback. This approach has the advantage of strong robustness to camera model deviations and requires less real-time computation compared to PBVS^20^. However, it also has limitations such as unknown depth information in the image Jacobian matrix, and which may encounter the singularity of the image Jacobian matrix and the problem of local minima^21^. Another approach is combination of both the IBVS and PBVS which is known as HVS method that improves the overall performance of a system selecting the best features among the two visual servoing techniques. HVS can combine the strengths of both approaches and benefited from depth information to achieve improved performance. The choice of visual servoing approach depends on the task requirements and camera availability^22^.

Visual servoing technique finds its application in object tracking. Identifying and continually monitoring objects across a series of picture or video frames is a key task in computer vision, known as object tracking^23,24^. This is performed by defining the target item in the first frame and then tracking it in the following frames^25^. Numerous fields have benefited from object trackings, such as activity recognition, robotics, autonomous vehicle tracking, traffic monitoring, and medical diagnosis systems^26,27^. Object tracking methods can be broadly divided into three categories: feature-based, segmentation-based, and estimation-based. Feature-based methods rely on identifying specific features of the object, such as colour, texture, or optical flow. Segmentation-based methods divide the image into regions and track the object by keeping track of its position in each segment. Estimation-based methods use mathematical models to predict the object’s position over time. Some common techniques within each of these categories include Bayesian, Kalman, and particle filters, as well as mean-shift and Cam Shift algorithms^26^. However, object tracking also poses significant challenges such as illumination variation, background clutters, low resolution, scale, occlusion, change in the target position and fast motion^28^ leading to ongoing research in this field.

## Related work

In recent years, with advancements in deep learning and camera technologies, vision-based robot control has seen a rapid growth, in both theoretical foundations and innovative applications. This brief review focuses on few latest developments and their applications, particularly in the context of robot control using visual information.

The use of visual information for controlling 2-DOF robotic arms has been widely explored in recent years, with various approaches leveraging image processing, deep learning (DL), and reinforcement learning (RL) to enhance robotic control. However, a critical analysis of existing works reveals limitations in their adaptability, accuracy, and real-time performance, which this study aims to address.

Several studies have focused on utilizing image-based parameters for robotic control. For instance, Sharma et al.^29^ employed two image parameters to enhance the observability and manipulability of a 2-DOF robotic arm, particularly in adjusting the active camera’s position and trajectory. Similarly, Cong et al.^30^ proposed a vision-based network control system (NCS) for remote manipulator control, integrating an image processing and transmission module. Wang et al.^31^ developed a low-cost, contactless trajectory tracking controller for a 2-DOF inverted pendulum, demonstrating the feasibility of vision-based control in robotic applications.

Advancements in sensor-based vision control have also been explored. Al-Shabi et al.^32^ utilized a Kinect sensor to extract image landmarks from skeletal data for robotic arm control. Moreno et al.^33^ introduced a machine vision-based system that interprets an operator’s arm movements to control a robotic arm, whereas Quintero et al.^34^ used 2D video images to drive a 6-DOF robot manipulator for assistive applications.

Deep learning and RL approaches have further improved robotic vision-based control. Athulya et al.^35^ applied a pre-trained model for inverse kinematics, using predefined joint-angle data for robotic control. Sekkat et al.^36^ introduced a deep reinforcement learning (DRL) based controller to determine an object’s 3D location for improved robotic arm control. Oliva et al.^37^ combined visual feedback with neural networks (NNs) to enhance control policy learning for a 2-DOF planar robot. Deng et al.^38^ proposed an active vision-based motion control technique, focusing on high-precision manipulation, while Yurtsever et al.^39^ analyzed vision-based deviation detection in a 2-DOF soft robotic arm. Additionally, Wang et al.^40^ introduced visual feature constraints to improve autonomous grasping tasks, and Belalia et al.^41^ demonstrated a CNN-based visual control framework for a 2-DOF SCARA arm through simulation studies.

While these approaches have made significant contributions, a comprehensive investigation into IBVS-based tracking control for a 2-DOF robotic arm remains unexplored. Existing works primarily focus on object detection, localization, and trajectory planning, but they lack an integrated real-time vision-based control mechanism that effectively leverages DL for improved precision and adaptability. This gap in the literature motivates the present study, which aims to develop an innovative IBVS framework for object tracking, integrating DL based feature extraction, dual 2D-3D coordinate utilization, and an optimized vision-based control scheme to enhance tracking accuracy and response efficiency.

This study defines the problem of achieving precise moving object tracking in a 2-DOF robotic arm and proposes a solution through the design of an interaction matrix using a minimal set of visual features extracted via a DL-based feature extraction method for enhanced visual control. The contributions of this presented study are as follows:

- Robust feature has been detected by utilizing deep learning-based object detection framework to address the issue associated with IBVS.
- A new vision-based control scheme is developed for tracking a moving object using a 2-DOF robotic arm, leveraging real-time responses from a deep learning-based object detection system to ensure high accuracy and fast response in visual servoing while maintaining simplicity and practicality.
- Developed the hardware and software interface to validate the performance of proposed control scheme using simulation and experimental study.
- Analyzed the safety aspects of the proposed vision-based control system.

This work introduces several key innovations in 2-DOF robotic arm control and computer vision, including the integration of MediaPipe for real-time feature extraction, a new dual-coordinate approach leveraging both 2D and 3D spatial information, and an optimized vision-based control scheme that enhances accuracy and efficiency in visual servoing tasks.

The importance of this work lies in the ability to enhance the flexibility of 2-DOF robotic arm by implementing a vision-based object tracking system, allowing robotic arms to adapt to environmental changes while reducing sensor dependency through the use of a single-lens camera and computer vision techniques, making the system more cost-effective and robust.

The rest of the paper is organised as follows. In section “Dynamics of 2-DOF robotic arm and camera modeling”, the dynamics of the 2-DOF robotic arm and camera model are described. Then, the selection of visual features for efficient tracking of moving objects is presented in section “Feature selection”, in which visual features for tracking control of 2-DOF (section “Visual features for tracking control of 2-DOF”) are presented followed by discussion of feature-based object detection (section “Feature based object recognition framework”). Section “Proposed vision-based tracking control scheme” presents the development of a proposed vision-based controller to track the moving object. Section “Results and discussion” presents the results and discussion, in which employed software and hardware components (section “Software and hardware components”) are described followed by comprehensive simulation results (section “Simulation results”) using CoppeliaSim simulator. Further, experimental results presented in section “Experimental results”. The conclusion and scope of further work are given in section “Conclusion and future scope”.

## Dynamics of 2-DOF robotic arm and camera modeling

### Dynamics of 2-DOF robotic arm

**Fig. 1:**
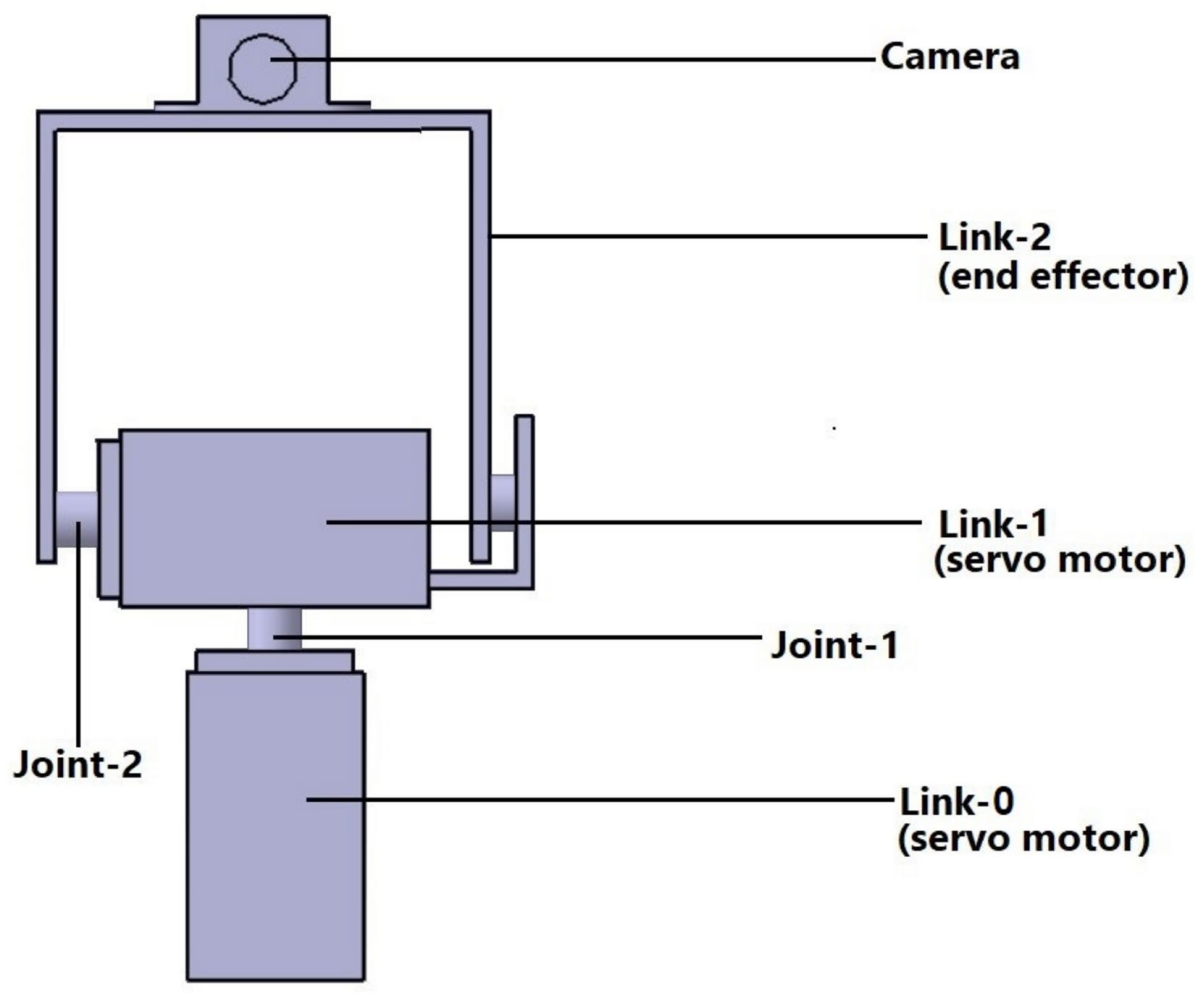
Structure of 2-DOF robotic arm.

**Fig. 2:**
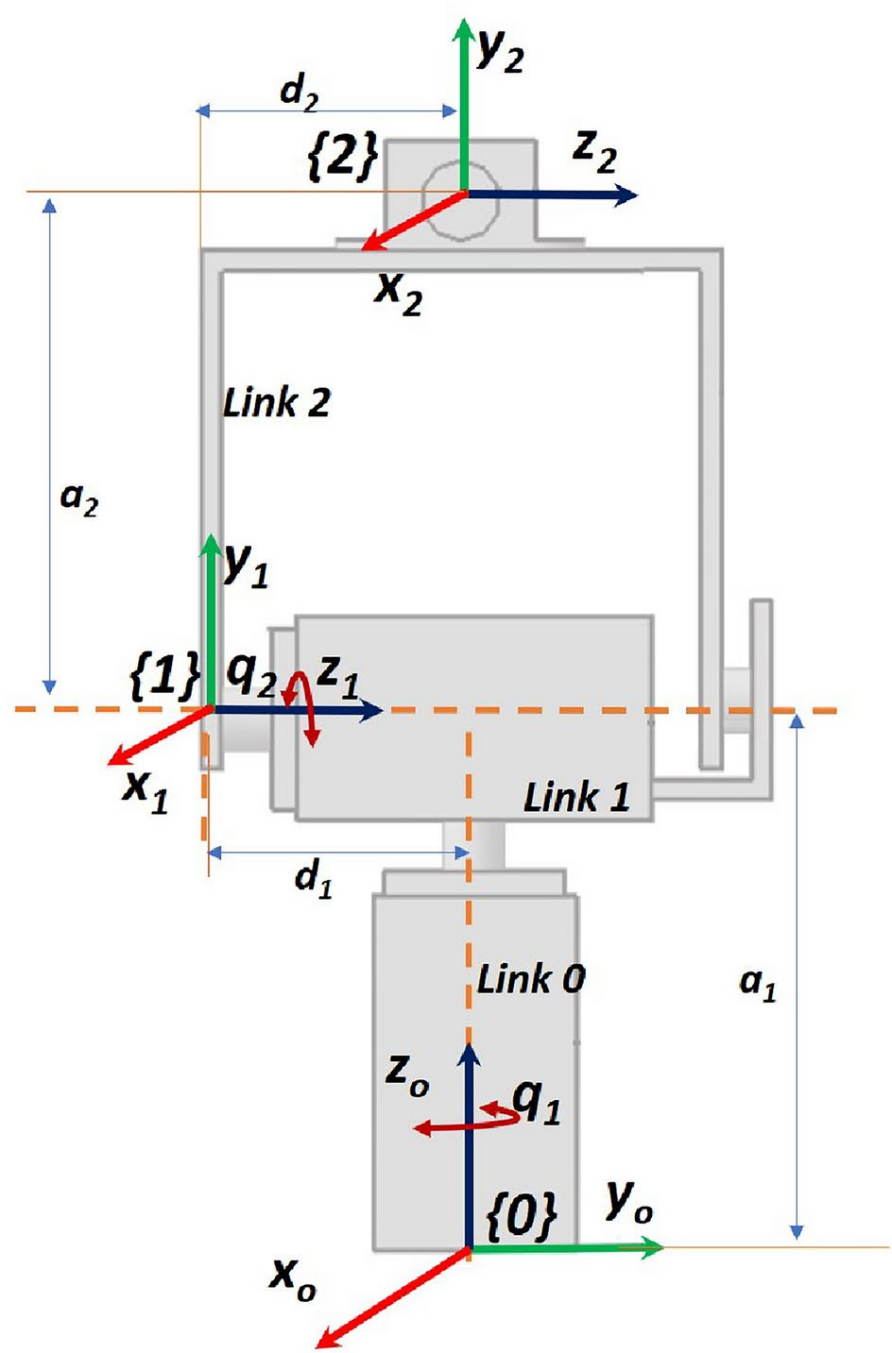
2-DOF robotic arm configuration.

The robotic arm consists of two revolute joints and the first two links are servo motors. First servo motor rotates from 0° to 180° along the vertical axis and the second servo motor rotates from 0° to 180° along the horizontal axis. The end-effector is camera placed in the U-shaped link as shown in the Fig. 1. The 2-DOF robot arm configuration is shown in the Fig. 2. The Denavit–Hartenberg (DH) parameters for the forward kinematics are given is Table 1.

**Table 1:** DH parameters for the forward kinematics.

Joint-1	
Link Length ($a_1$)	2.3 inches (length from base to Joint-1.)
Link Twist ($\alpha _1$)	90° (since $z_1$ and $z_2$ are perpendicular)
Link Offset ($d_1$)	1.15 inches (distance from joint-1 to joint-2, along z-axis)
Joint Angle Offset ($q_1$)	Rotates about $z_0$ (pan movement)
Joint-2	
Link Length ($a_2$)	2.1 inches (length from Joint-2 to the end-effector.)
Link Twist ($\alpha _2$)	0° (since $z_1$ and $z_2$ are parallel)
Link Offset ($d_2$)	1.15 inches (distance from joint-2 to end-effector, along z-axis)
Joint Angle Offset ($q_2$)	Rotates about $z_1$ (tilt movement)

Based on the DH parameters, the transformation matrix from the base frame to the end-effector frame can be derived using the DH convention. The transformation matrix, denoted as *T*, is obtained by multiplying individual transformation matrices for each joint can be expressed as:

1 $$\begin{aligned} T = T_1 \times T_2 \end{aligned}$$

where $T_1$ and $T_2$ represent the individual transformation matrices for each joint is described in Eqs. (2) and (3).

2 $$\begin{aligned} & {T_1} = \left[ {\begin{array}{*{20}{c}} {\cos {q_1}}& 0 \quad & \sin {q_1} & \quad 2.3\cos {q_1}\\ {\sin {q_1}}& 0 \quad & {-\cos {q_1}}& \quad 2.3\sin {q_1}\\ 0& \quad 1& \quad 0& \quad 1.15\\ 0& \quad 0& \quad 0& \quad 1 \end{array}} \right] \end{aligned}$$

3 $$\begin{aligned} & {T_2} = \left[ {\begin{array}{*{20}{c}} {\cos {q_2}}& \quad {-\sin {q_2}}& \quad 0& \quad 2.1\cos {q_2} \\ { \sin {q_2}}& \quad {\cos {q_2}}& \quad 0& \quad 2.1\sin {q_2} \\ 0& \quad 0& \quad 1& \quad 1.15 \\ 0& \quad 0& \quad 0& \quad 1 \end{array}} \right] \end{aligned}$$

The resulting matrix *T* given in (4) represents the transformation from the base frame to the end-effector frame. The elements in the matrix correspond to the position and orientation of the end-effector relative to the base frame.

4 $$\begin{aligned} T = \left[ {\begin{array}{*{20}{c}} {\cos {q_1} \cos {q_2}}& \quad { -\cos {q_1}\sin {q_2}}& \quad {\sin {q_1}}& \quad 2.3\cos {q_1}+2.1\cos {q_1}\cos {q_2}+1.15\sin {q_1}\\ {\sin {q_1} \cos {q_2}}& \quad {-\sin {q_1} \sin {q_2}}& \quad {-\cos {q_1}}& \quad 2.3\sin {q_1}+2.1\sin {q_1}\cos {q_2}-1.15\cos {q_1} \\ { \sin {q_2}}& \quad {\cos {q_2}}& \quad 0& \quad 2.1 \sin {q_2}+1.15 \\ 0& \quad 0& \quad 0& \quad 1 \end{array}} \right] \end{aligned}$$

By controlling and adjusting the joint angles $q_1$ and $q_2$, camera can be positioned accordingly to track the object’s movement.

### Camera modeling

For a 2-DOF based robotic arm, the interaction matrix for visual servoing with point features must depend on the specific configuration of the robot arm and its arm kinematics. The robotic arm has two joints. These joints are denoted by joint-1 and joint-2 as described in Fig. 1. Moreover, the joint angles are represented as $q_1$ and $q_2$, respectively. Further, the relationship between the joint velocities and the changes in the image coordinates of the point feature are formulated to compute the interaction matrix. This derived relationship is typically based on the forward kinematics of the robot arm, which describes about joint angles in determination of position of the end-effector in Cartesian coordinates. The image coordinates in terms of Cartesian coordinates provides the relation between image coordinates and its reference joint angles by employing chain rule of differentiation. This involves calculating the partial derivatives of the image coordinates with respect to the Cartesian coordinates and then with respect to joint angles. The interaction matrix, denoted as *L*, for a 2-DOF robot arm, can be represented as

5 $$\begin{aligned} L = \left[ {\begin{array}{*{20}{c}} {\frac{{du}}{{d{q_1}}}}& \quad {\frac{{du}}{{d{q_2}}}} \\ {\frac{{dv}}{{d{q_1}}}}& \quad {\frac{{dv}}{{d{q_2}}}} \end{array}} \right] \end{aligned}$$

Here, $\frac{{du}}{{d{q_1}}}$, and $\frac{{du}}{{d{q_2}}}$, respectively, represent the partial derivatives of the image coordinate *u* with respect to joint angles $q_1$ and $q_2$. Similarly, $\frac{{dv}}{{d{q_1}}}$ represent the partial derivatives of the image coordinate *v* with respect to the joint angles $q_1$, and partial derivatives of the image coordinate *v* with respect to the joint angles $q_2$ is given by $\frac{{dv}}{{d{q_2}}}$.

The specific values of these partial derivatives will depend on the geometry and kinematics of the robotic arm. So, these partial derivatives based on the forward kinematics equations that relate the joint angles to the Cartesian coordinates of the end-effector and the image coordinates of the point feature needs to be derived. The interaction matrix (5), is used in visual servoing algorithms to determine the required joint velocities that will drive the robotic arm to achieve a desired change in the image coordinates of the point feature.

Let’s denote the Cartesian coordinates of the end-effector as (*X*, *Y*) and the image coordinates of the point feature as (*u*, *v*) . The forward kinematics equations of the 2-DOF robotic arm will define how the end-effector position relates to the joint angles. Let’s assume these equations are as follows:

6 $$\begin{aligned} \begin{aligned}&X = {f_1}({q_1},{q_2}) \\&Y = {f_2}({q_1},{q_2}) \\ \end{aligned} \end{aligned}$$

Equation (6) is a general representation of the forward kinematice, presenting the *X* and *Y* coordinates of the end-effector in terms of the joint angles. Where, $f_1$ and $f_2$ are derived from last column of the DH transformation matrix derived in (4).

Now, the partial derivatives of the image coordinates (*u*, *v*) with respect to the joint angles $(q_1, q_2)$ using the chain rule of differentiation can be represent as:

7 $$\begin{aligned} \left\{ \begin{aligned} \frac{{du}}{{d{q_1}}} = \frac{{du}}{{dX}}*\frac{{dX}}{{d{q_1}}} + \frac{{du}}{{dY}}*\frac{{dY}}{{d{q_1}}} \\ \frac{{du}}{{d{q_2}}} = \frac{{du}}{{dX}}*\frac{{dX}}{{d{q_2}}} + \frac{{du}}{{dY}}*\frac{{dY}}{{d{q_2}}} \\ \frac{{dv}}{{d{q_1}}} = \frac{{dv}}{{dX}}*\frac{{dX}}{{d{q_1}}} + \frac{{dv}}{{dY}}*\frac{{dY}}{{d{q_1}}} \\ \frac{{dv}}{{d{q_2}}} = \frac{{dv}}{{dX}}*\frac{{dX}}{{d{q_2}}} + \frac{{dv}}{{dY}}*\frac{{dY}}{{d{q_2}}} \\ \end{aligned} \right\} \end{aligned}$$

where $\frac{{du}}{{d{X}}}$, $\frac{{du}}{{d{Y}}}$, $\frac{{dv}}{{d{X}}}$, $\frac{{dv}}{{d{Y}}}$ represent the partial derivatives of the image coordinates (*u*, *v*) with respect to the Cartesian coordinates (*X*, *Y*) of the end-effector. These are then combined with the partial derivatives of the Cartesian coordinates with respect to the joint angles to form the interaction matrix. The terms $\frac{{dX}}{{d{q_1}}}$, $\frac{{dX}}{{d{q_2}}}$, $\frac{{dY}}{{d{q_1}}}$, $\frac{{dY}}{{d{q_2}}}$ represent the partial derivatives of the Cartesian coordinates (*X*, *Y*) with respect to the joint angles $(q_1, q_2)$.

From (4), Eq. (6) can be represented as

8 $$\begin{aligned} & X = a_1\cos {q_1}+a_2\cos {q_1}\cos {q_2}+d_2\sin {q_1} \end{aligned}$$

9 $$\begin{aligned} & Y = a_1\sin {q_1}+a_2\sin {q_1}\cos {q_2}-d_2\cos {q_1} \end{aligned}$$

Differentiation of (8) with respect to $q_1$ and $q_2$ can be represented as

10 $$\begin{aligned} \begin{aligned}&\frac{{dX}}{{d{q_1}}} = - {a_1}\sin {q_1}- {a_2}\sin {q_1}\cos {q_2}+d_2\cos {q_1} \\&\frac{{dX}}{{d{q_2}}} = - {a_2}\cos {q_1}\sin {q_2} \\ \end{aligned} \end{aligned}$$

Differentiation of (9) with respect to $q_1$ and $q_2$ can be represented as

11 $$\begin{aligned} \begin{aligned}&\frac{{dY}}{{d{q_1}}} = {a_1}\cos {q_1}+ {a_2}\cos {q_1}\cos {q_2}+d_2\sin {q_1} \\&\frac{{dY}}{{d{q_2}}} = -{a_2}\sin {q_1}\sin {q_2} \\ \end{aligned} \end{aligned}$$

In (7), $\frac{{du}}{{d{X}}}$, $\frac{{du}}{{d{Y}}}$, $\frac{{dv}}{{d{X}}}$, $\frac{{dv}}{{d{Y}}}$ are derived from perspective projection camera model, for mapping real-world 3D position into pixel coordinate (*u*, *v*). by following perspective projection camera model^20^:

12 $$\begin{aligned} \begin{aligned} u=\frac{fX}{Z}; v= \frac{fY}{Z} \end{aligned} \end{aligned}$$

where, *f* is the focal length of camera, and (*X*, *Y*, *Z*) is the position of the end-effector in the camera frame. We assume the end-effector lies on a plane with a fixed depth *Z*. Derivative of perspective projection (12) can be written as

13 $$\begin{aligned} \begin{aligned} \frac{du}{dX} = \frac{f}{Z}; \frac{du}{dY}= 0 ; \frac{dv}{dX}= 0 ; \frac{dv}{dY}= \frac{f}{Z}. \end{aligned} \end{aligned}$$

Substituting (10), (11), and (13) into (7), interaction matrix (5) can be represented as

14 $$\begin{aligned} L = \left[ {\begin{array}{*{20}{c}} - f({a_1}\sin {q_1}+ {a_2}\sin {q_1}\cos {q_2}-d_2\cos {q_1})/Z & - f({a_2}\cos {q_1}\cos {q_2})/Z \\ f({a_1}\cos {q_1}+ {a_2}\cos {q_1}\cos {q_2}+d_2\sin {q_1})/Z & - f({a_2}\sin {q_1}\sin {q_2})/Z & \end{array}} \right] \end{aligned}$$

Equation (14) is the interaction matrix that relates the joint velocities to the changes in the image coordinates (*u*, *v*) of the point feature. It captures the sensitivity of the image coordinates to the joint angles. Further, the joint angles are used in visual servoing algorithms to control the robot arm based on the desired changes in the image coordinates.

## Feature selection

### Visual features for tracking control of 2-DOF

In this section, the implementation of visual feature extraction and control for a 2-DOF robotic arm to track specific landmarks are done. The chosen landmark for this task is a person’s nose tip, which is tracked using IBVS. The process begins with using MediaPipe, a computer vision framework, to detect the position of the moving object in real-time from a video stream. MediaPipe’s pose estimation model predicts the 2D coordinates of the moving object, which are then transformed into 3D world coordinates through camera calibration. These 3D coordinates serve as the visual feature for controlling the robotic arm. A visual servoing control loop calculates the required joint angles of the robotic arm based on the error between the current position and the desired position using (14). The calculated joint angles are then used to actuate the robotic arm, enabling it to accurately track the moving object as it moves within the video stream. This approach allows for precise and dynamic object tracking with the robotic arm.

To map 2D image coordinates (*u*, *v*) to 3D world coordinates (*X*, *Y*, *Z*) for the given robotic arm with DH parameters (Table 1), camera calibration need to be perform to obtain the intrinsic parameters of the camera and then use the forward kinematics equations for the robotic arm to compute the 3D position. Here are the steps to achieve this:

#### Camera calibration

Camera calibration is a crucial step to estimate the intrinsic parameters of the camera. The important parameters involved in camera calibration are focal lengths $(f_x, f_y)$, principal point coordinates $(c_x, c_y)$, and lens distortion parameters $(k_1, k_2, k_3)$. In this process, firstly, by identifying the known calibration patterns, such as a checkerboard, to capture multiple images, analyzing is done. By analyzing the images, the intrinsic parameters of the camera can be estimated, accurately. The camera calibration parameters are also essential for transforming 2D image coordinates into 3D world coordinates, as such parameters define the camera’s internal characteristics to capture the visual information.

#### Obtain 2D image coordinates

After camera calibration, the next step is to extract the 2D image coordinates of a feature point from the captured camera image. This is achieved using MediaPipe, which detects specific landmarks, such as the nose tip, and provides their 2D coordinates (*u*, *v*) . These coordinates serve as a crucial input for subsequent transformations into 3D coordinates.

#### Convert to homogeneous coordinates

To facilitate the transformation from 2D to 3D coordinates, the 2D image coordinates need to be represented in homogeneous form. This is done by adding a 1 as the third component to the coordinates (*u*, *v*) , resulting in homogeneous coordinates $(u', v', 1)$. This representation is necessary for applying the inverse projection and other mathematical transformations.

#### Inverse projection

The inverse projection step involves obtaining the direction of the ray from the camera’s optical center in the 3D world frame. This is achieved by applying the inverse of the camera’s intrinsic matrix (*L*) to the homogeneous coordinates $(u', v', 1)$. The result is a set of coordinates that represent the direction of the ray in the 3D world frame. This step is crucial for determining the 3D position of the point feature relative to the camera.

#### Compute 3D point

Finally, the 3D position of the end-effector/camera in the world coordinate system is computed using the transformation matrix (*T*) derived from the DH parameters. The forward kinematics equations and the transformation matrix are used to calculate the 3D coordinates (*X*, *Y*, *Z*) of the end-effector. The transformation matrix *T* represents the position and orientation of the end-effector relative to the base frame. By extracting the 3D coordinates from this matrix, the position of the end-effector in the world coordinate system can be determined. These values correspond to the position of the end-effector after setting the joint angles $q_1$ and $q_2$ in the robotic arm. By adjusting these joint angles, the camera /end-effector can be positioned to track the movement of the object accurately.

### Feature based object recognition framework

In the present work, a MediaPipe framework is used as feature based object recognition framework. The presented framework is utilized to extract the point feature, and will be tracking the movement of the facial landmark. The process begins with a pre-trained machine-learning model extensively trained on diverse datasets that recognize intricate patterns. Through feature extraction, the neural network identifies key points representing various facial features, which localizes the object point in 2D image coordinates (*u*, *v*) . Once MediaPipe processes the input, it outputs the facial landmark information, which includes the object coordinates, OpenCV provide a real time monitoring and pre-process the input from the camera such as resizing, and colour combination with respect to the compatibility of the object point.

#### Camera calibration and coordinate transformation

Subsequently, camera calibration becomes crucial to convert the 2D coordinates to the camera frame and determine intrinsic parameters like focal lengths $(f_x, f_y)$ and principal point coordinates $(c_x, c_y)$. The 2D image coordinates are then converted to homogeneous coordinates $[u', v', 1]$ for further processing. By utilizing the inverse of the camera’s intrinsic matrix $L^{-1}$ the direction of the ray from the camera’s optical center in 3D world coordinates is obtained. The last step would involve using the 3D vector $[u, v, 1]^T$ and the depth information to compute the actual 3D position (*X*, *Y*, *Z*) of the object point in the world coordinate system.

#### Detailed process of object recognition framework

**Fig. 3:**
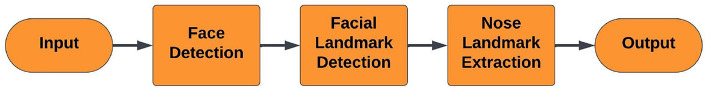
MediaPipe moving object point extraction flowchart.

MediaPipe systematically extracts object features through a well-defined sequence of steps outlined below in Fig. 3.

Initially, the system ingests video frames or images. Face detection is then executed using the Single Shot Multibox Detector framework, known for its efficiency in real-time object detection. Following this, the facial landmarks detection step employs a custom neural network with the “Holistic-Model” technique. This specialized algorithm accurately identifies numerous critical points on the face. The next step is to extract the moving object landmark, which involves accurately locating the corresponding key point within the output of the face landmark detection. This extraction technique is tightly linked to the overall architecture of the holistic model. Finally, the system generates an output with the specific coordinates of the detected moving object^42^. MediaPipe’s output includes detailed facial landmark information and accurate coordinates for significant points, particularly those linked with the object.

#### Integration with OpenCV

Following the extraction of object features, OpenCV plays a crucial role in enhancing the workflow by enabling further processing steps. This includes tasks such as creating visual representations, performing additional analytical operations, and seamlessly integrating the collected object detection insights into larger and more advanced computer vision systems. The combination of MediaPipe with OpenCV exemplifies a successful collaboration, demonstrating the integration of DL capabilities for refined object detection in computer vision applications^43^.

#### Application in robotic arm control

Integrating this process with the control of a 2-DOF robotic arm, the extracted 2D coordinates from MediaPipe are transformed into 3D world coordinates through camera calibration. These 3D coordinates serve as the visual feature for controlling the robotic arm. A visual servoing control loop calculates the required joint angles of the robotic arm based on the error between the current position and the desired position. The calculated joint angles are then used to actuate the robotic arm, enabling it to accurately track the moving object as it moves within the video stream. In our control logic, we use the 3D coordinates primarily to control the speed in the *Y* direction. However, for controlling the two robotic arm joints, we rely only on the 2D points. This approach allows for efficient and precise control of the robotic arm’s movements, ensuring accurate tracking of the moving object. By following these detailed steps, the system can dynamically and precisely track a moving object, leveraging the strengths of both MediaPipe and OpenCV in a cohesive and effective manner.

## Proposed vision-based tracking control scheme

**Fig. 4:**
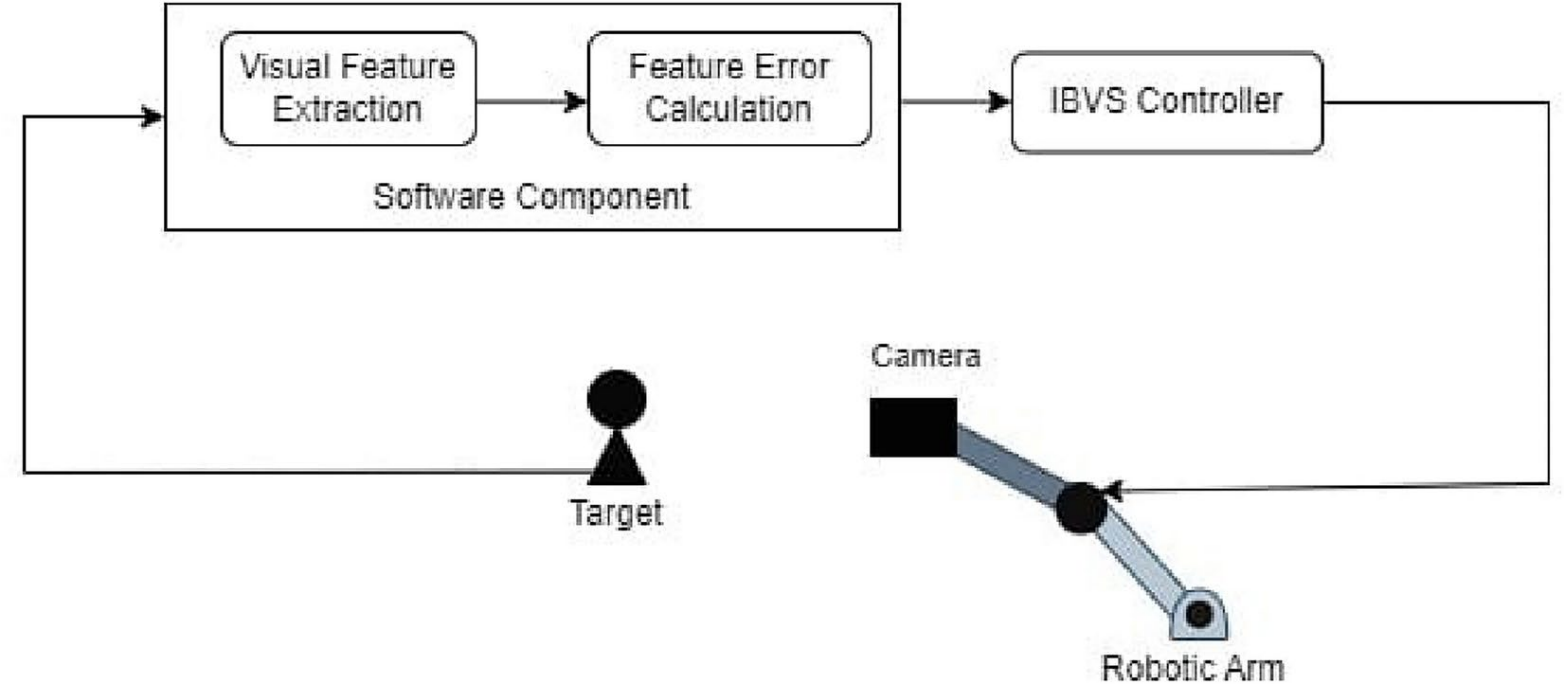
Block diagram of IBVS tracking control system.

The IBVS tracking control system follows a systematic procedure. It starts by utilizing a camera to monitor the real-time position of an object, as shown in Fig. 4. Subsequently, the system calculates the positional error by comparing it with the end effector’s position. Utilizing this error data, the controller then generates control commands for the joints of 2-DOF robotic arms. These drives promptly respond by adjusting the joint angles, a critical step to minimize the error and attain the desired end-effector position.

In proposed vision-based tracking control scheme the goal is to control the 2-dof robotic arm so that the image coordinates (*u*, *v*) matches the desired image coordinates $(u^*, v^*)$. This is achieved by inverse kinematics given below

15 $$\begin{aligned} \dot{q} = {L^ + }\left[ {\begin{array}{*{20}{c}} {\dot{u}} \\ {\dot{v}} \end{array}} \right] \end{aligned}$$

where $L^+$ is the pseudo-inverse of the interaction/ Jacobin matrix (14), $\dot{u}$ and $\dot{v}$ are the required changes in image coordinates, and $\dot{q}$ is the joint velocity command. To move the robot towards a moving target in the image the error between desired and current image coordinates can be computed using,

16 $$\begin{aligned} e = \left[ {\begin{array}{*{20}{c}} {{u^*} - u} \\ {{v^*} - v} \end{array}} \right] \end{aligned}$$

The control law can be defined as

17 $$\begin{aligned} \left[ {\begin{array}{*{20}{c}} {\dot{u}} \\ {\dot{v}} \end{array}} \right] = - \lambda e \end{aligned}$$

where, $\lambda$ is a positive gain. Using (15), joint velocities can be computed using

18 $$\begin{aligned} \dot{q} = {L^ + }( - \lambda e) \end{aligned}$$

**Fig. 5:**
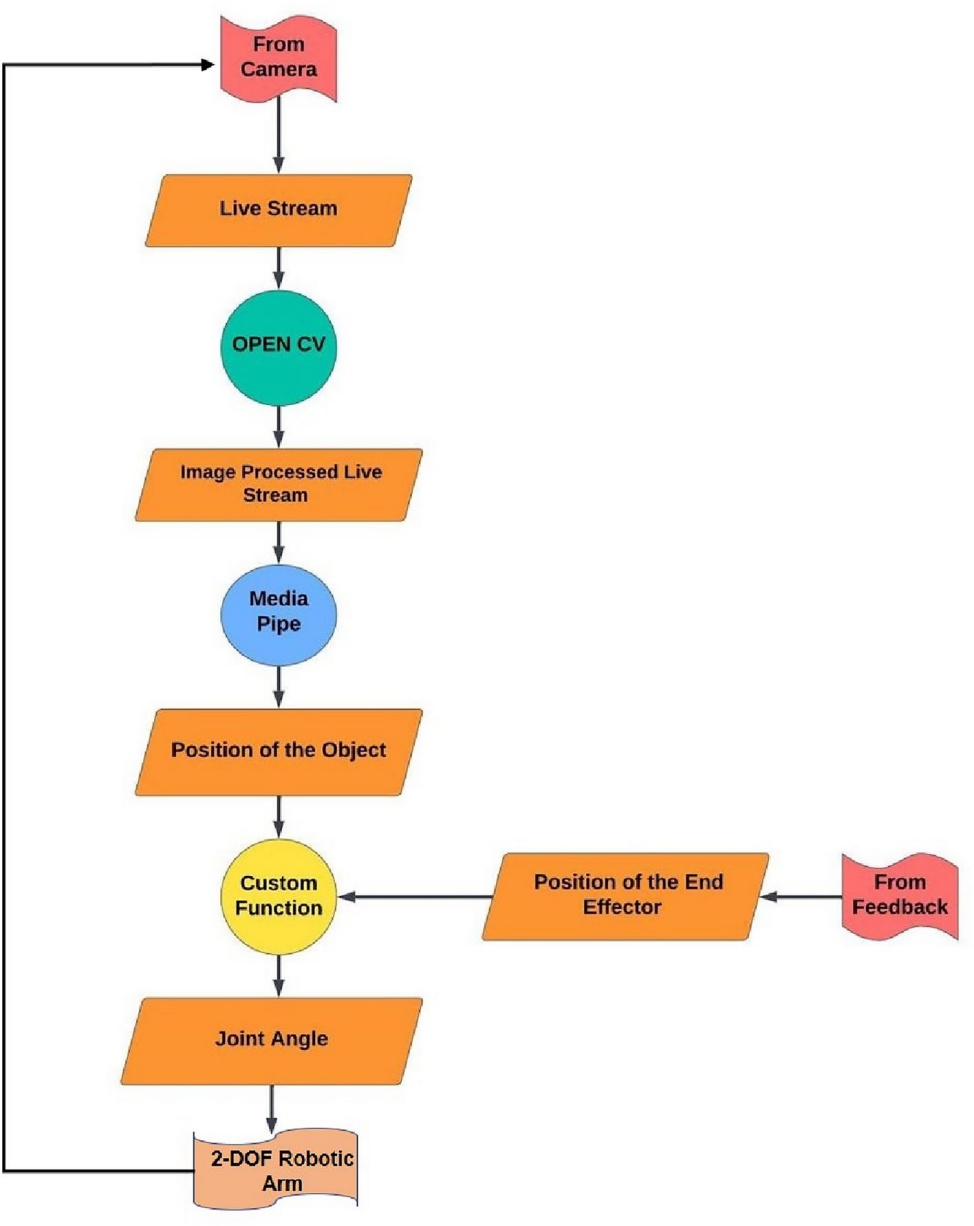
Stepwise explanation of IBVS tracking process.

Equation (18), updates joint position by integrating $\dot{q}$ over time. In Fig. 5 shown the proposed IBVS controller, in which the process to track the moving object consisting of three main stages. Initially, the camera is observing the moving object. The visual feature from the moving object is extracted by feature-based object detection framework (section “Feature based object recognition framework”) in later stage. Incorporating these extracted visual features and desired feature, feature error is determined. The third stage employs an IBVS control algorithm to compute the necessary joint angles for achieving the desired position. This algorithm strategically divides the field of view into specific sections, assigning each section with corresponding joint angles. Thereby minimizing the error between the object and the end-effector position, bringing it to zero. The entire process is visually depicted in Fig. 5.

The IBVS tracking control scheme shown in Fig. 4 consists of a hardware and a software component. The software component MediaPipe framework is used as feature-based object recognition framework, which observe the moving object from the camera to detect the object’s position. Subsequently, it calculates the necessary joint angles from the feature error to attain that position and sends commands from the controller designed using ATmega328P based development board to control the joints of the 2-DOF robotic arm. The simulation and experimental studies are carried out and presented in section “Results and discussion” to validate the proposed IBVS tracking contol scheme.

### Stability analysis

To analyze stability, define a Lyapunov function candidate:

19 $$\begin{aligned} V=\frac{1}{2}e^Te \end{aligned}$$

which represents the squared image feature error. Time derivative of (19) can be written as

20 $$\begin{aligned} \dot{V}=e^T \dot{e} \end{aligned}$$

Using (18), the image feature dynamics are given by $\dot{e} = -LL^ + \lambda e$. Above Eq. (20) can be represented as.

21 $$\begin{aligned} \dot{V}=-\lambda e^T LL^ + e \end{aligned}$$

Since, $LL^+$ is semi-positive definite, and $\lambda$ is positive, we conclude the $\dot{V} \le 0$, which ensure that V is non-increasing. This implies global asymptotic stability, meaning the system will converge to the desired image coordinates. Also, if $\lambda$ is large, convergence is fast, and if $\lambda$ is small, convergence is slow. This ensures that the robot smoothly tracks the moving target.

## Results and discussion

In this section, the performance of the proposed IBVS tracking controller has been analyzed by simulation and experimental studies. The simulation analysis of the proposed controller is evaluated in the CoppeliaSim simulator^44^. Further, the development of the experimental setup is presented followed by the performance and robustness of the proposed IBVS tracking controller is validated by performing experimental studies on the 2-DOF Robotic arm.

### Software and hardware components

This section outlines the hardware and software components utilized in both, simulation and experimental studies. The software components include the CoppeliaSim simulator, MediaPipe framework, OpenCV library and Arduino Integrated Development Environment (IDE). The hardware components of the experimental setup consists of a 2-DOF robotics arm, an ATmega328P-based controller, and a personal computer (PC). The detailed description of software and hardware components are presented in Tables 2 and 3, respectively.

**Table 2:** Software tools.

Software tools	Description
CoppeliaSim Simulator	The CoppeliaSim Simulator is an open-source simulator utilize for vision-based tracking of a 2-DOF robot arm. It also offers a versatile research environment, an accurate dynamic model, and interaction with ViSP for vision-based tracking control of a 2-DOF robotic arm
OpenCV library	The OpenCV library is one of the well known open-source machine vision library. It detects the outlines and center of an item on a workstation. Additionally, it also provides several functions for machine vision algorithms, including contouring, Canny Edge, grayscale, and Gaussian filter
MediaPipe framework	The MediaPipe is an open-source framework which is generally used to perform real-time perception tasks including object detection and tracking. MediaPipe is coupled and configured with OpenCV to improve the feature extraction for better vision-based tracking control. By utilizing OpenCV and MediaPipe, a system can be created to recognize and track objects in real-time which allows for precise control of a 2-DOF robot arm
Arduino IDE	The Arduino IDE is utilized for vision-based tracking control of a 2-DOF robot arm by integrating computer vision algorithms. It utilizes a vision system with IBVS. The main advantages of Arduino IDE are convenient operation, fast analyzing rate, stable operation, high accuracy, etc.

**Table 3:** Hardware components.

Hardware components	Description
2-DOF robotic arm	A 2-DOF robotic arm is applied for object tracking. This specialized arm, equipped with a camera on its end effector, accurately observes object movements in both horizontal and vertical directions. The camera facilitates smooth and uninterrupted tracking of the object, highlighting the indispensable nature of the 2-DOF robotic arm as a fundamental hardware component in our system
ATmega328P based Controller	The control system incorporates an Arduino UNO a ATmega328P based Development Board, recognized for its open-source architecture and user-friendly design. Arduino Uno is the key component in vision-based tracking control of a 2-DOF robot arm include integration of computer vision, vision-based control strategy, and enhanced performance^45^
Personal computer (PC)	It is equipped with Intel(R) Xeon(R) E-2124 processor operates at 3.30GHz clock cycle. PC is used to execute IBVS control algorithm using OpenCV, MediaPipe and facilitate integration of software tool with hardware components to achieve visual servoing task

### Simulation results

**Fig. 6:**
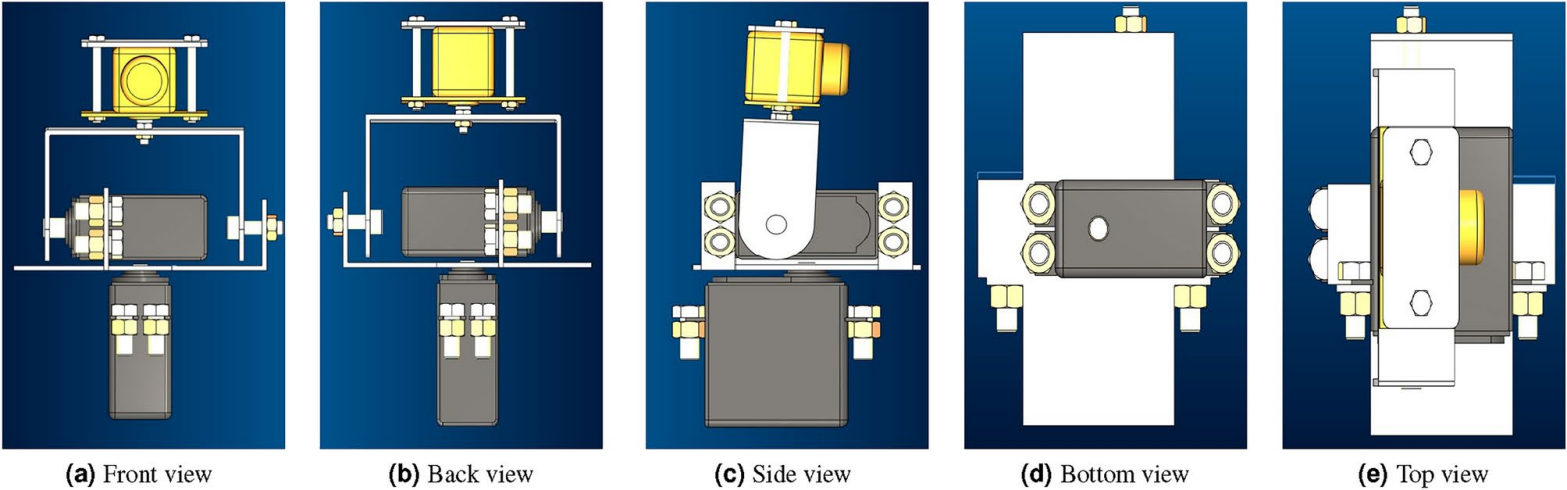
3D CAD model of 2-DOF robotic arm.

In this study, a comprehensive simulation was conducted utilizing the CoppeliaSim simulator^44^ to replicate the dynamics of a real-world system, specifically a 2-DOF robotic arm designed to track the movement of an object. In the present study, a sphere is considered an object that resembles a nose tip. Figure 6 shows the 3D Computer-aided design (CAD) model of the 2-DOF robotic arm with different views. In the present simulation study, two revolute joints were implemented in the simulated environment to mimic the mechanical structure of the 2-DOF robotic arm shown in Fig. 6. The IBVS tracking control algorithm implemented using OpenCV and MediaPipe successfully generated real-time *x* and *y* coordinate values for the object’s movement along a predefined path. By implementing a IBVS tracking algorithm, the simulated robotic arm effectively followed the object’s trajectory. The real-time tracking of the object’s coordinates and the adherence to the predefined path showcase the robustness and accuracy of the tracking algorithm within the CoppeliaSim environment.

**Fig. 7:**
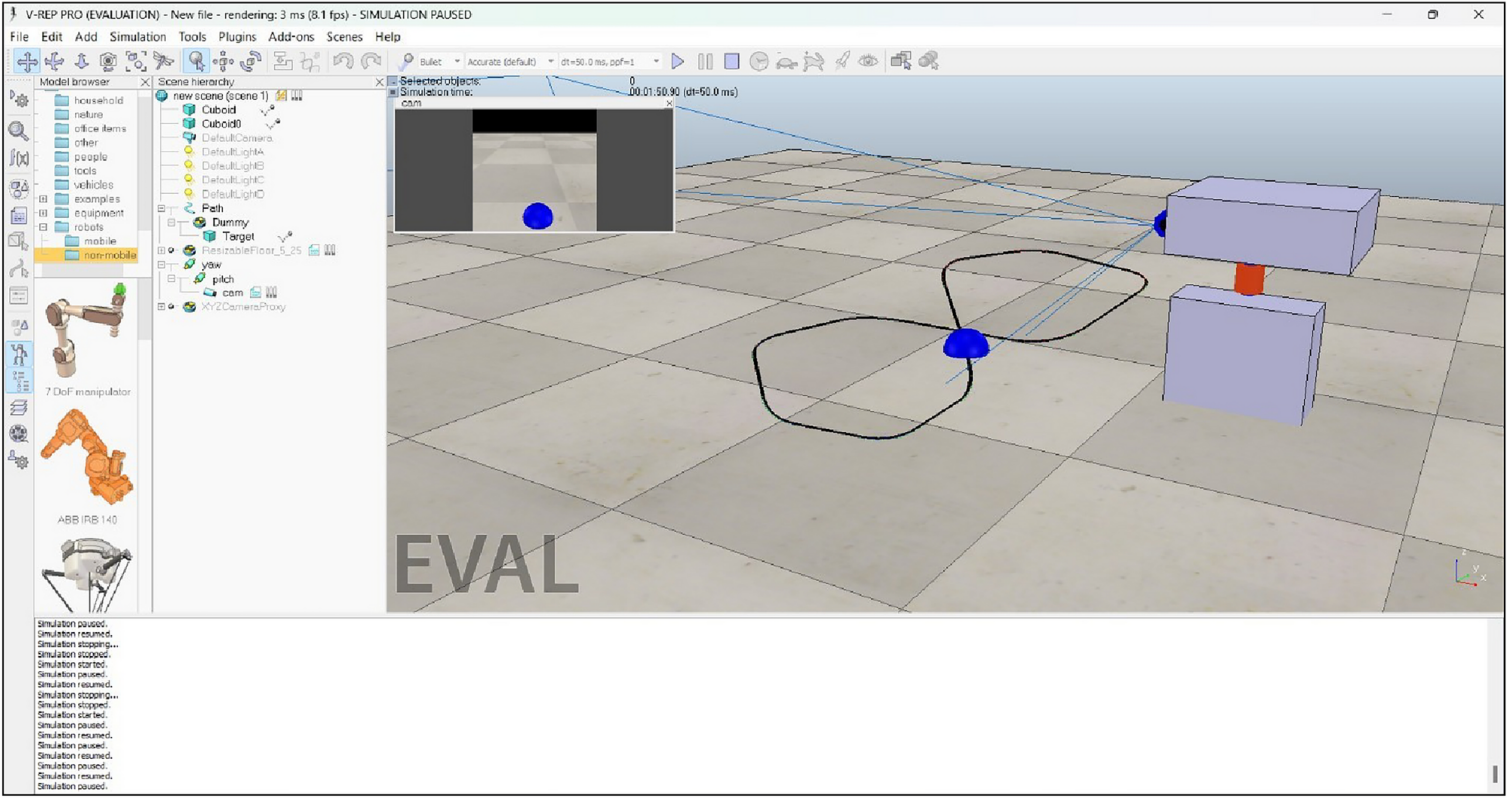
Path, object and 2-DOF robotic arm developed in CoppeliaSim environment.

In the CoppeliaSim environment, a random path has been generated alongside a spherical object resembling a nose tip. Accompanying this setup are two revolute joints representing the structure of a 2-DOF robotic arm, as depicted in Fig. 7.

**Fig. 8:**
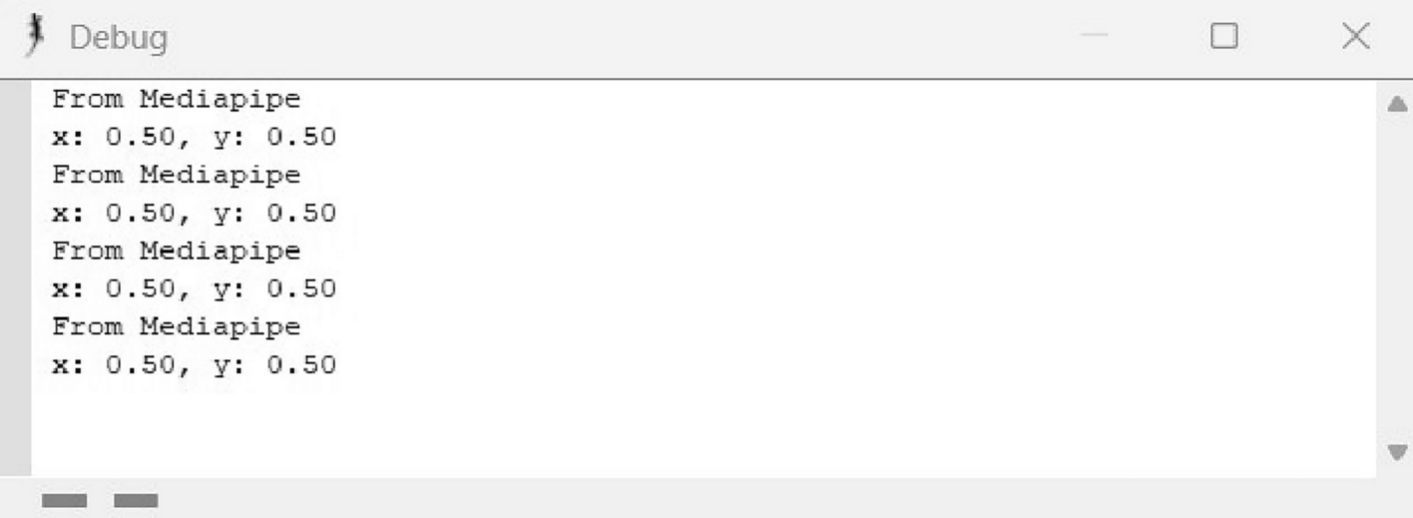
CoppeliaSim console showing x and y coordinate values of the object.

In the IBVS tracking algorithm implementation, MediaPipe and OpenCV libraries were utilized to capture real-time positional data of the object. The *x* and *y* coordinate values of the object were extracted, as shown in Fig. 8. Following this, the data was transmitted to the CoppeliaSim debug console via a Remote API connection established between VSCode and CoppeliaSim. This integration facilitates a structured exchange of information between the Python environment and the CoppeliaSim simulation, enabling seamless updates and interactions based on the live data acquired through object tracking.

**Fig. 9:**
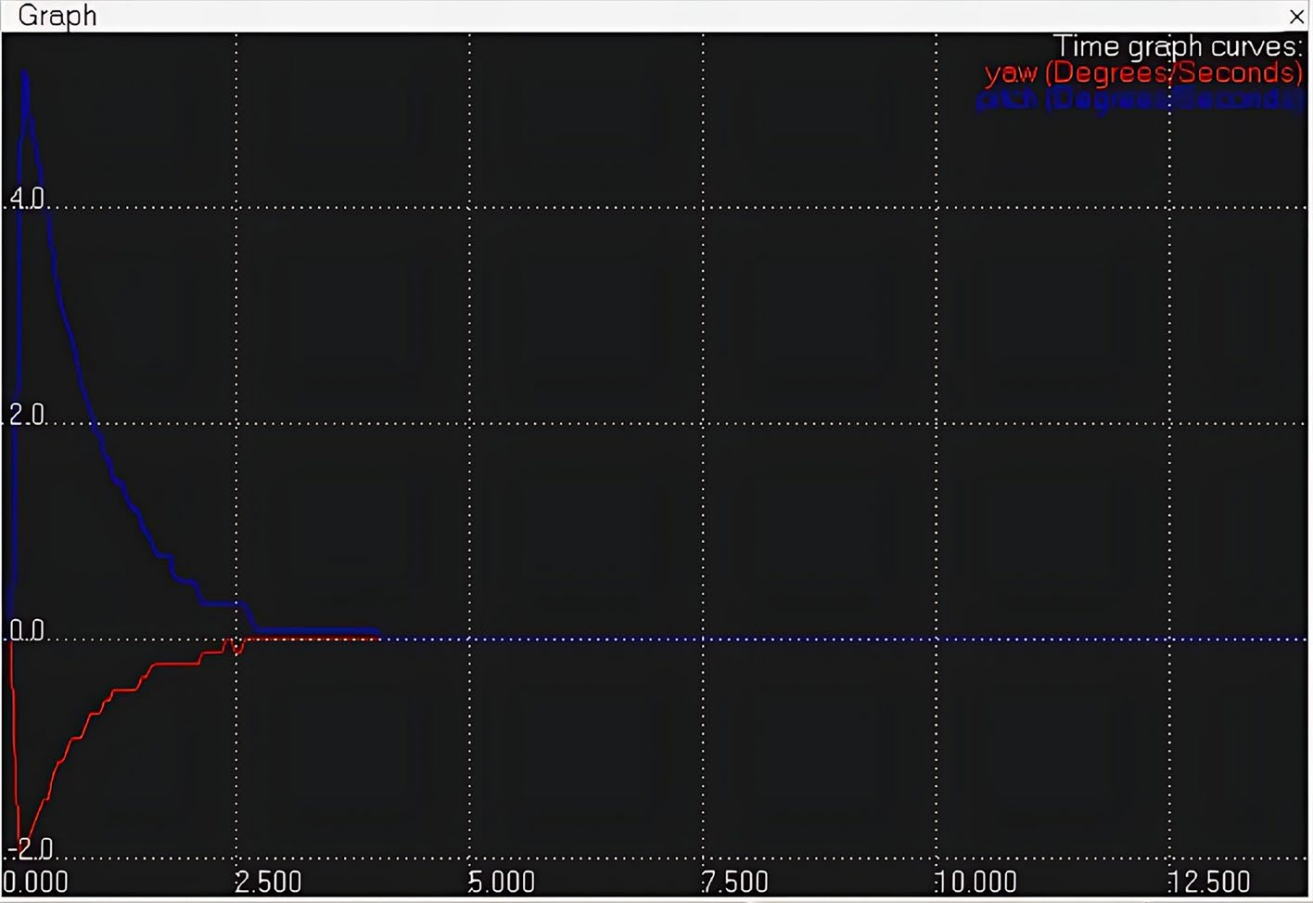
Plot of joint velocity v/s time (red indicates yaw velocity and blue indicates pitch velocity).

**Fig. 10:**
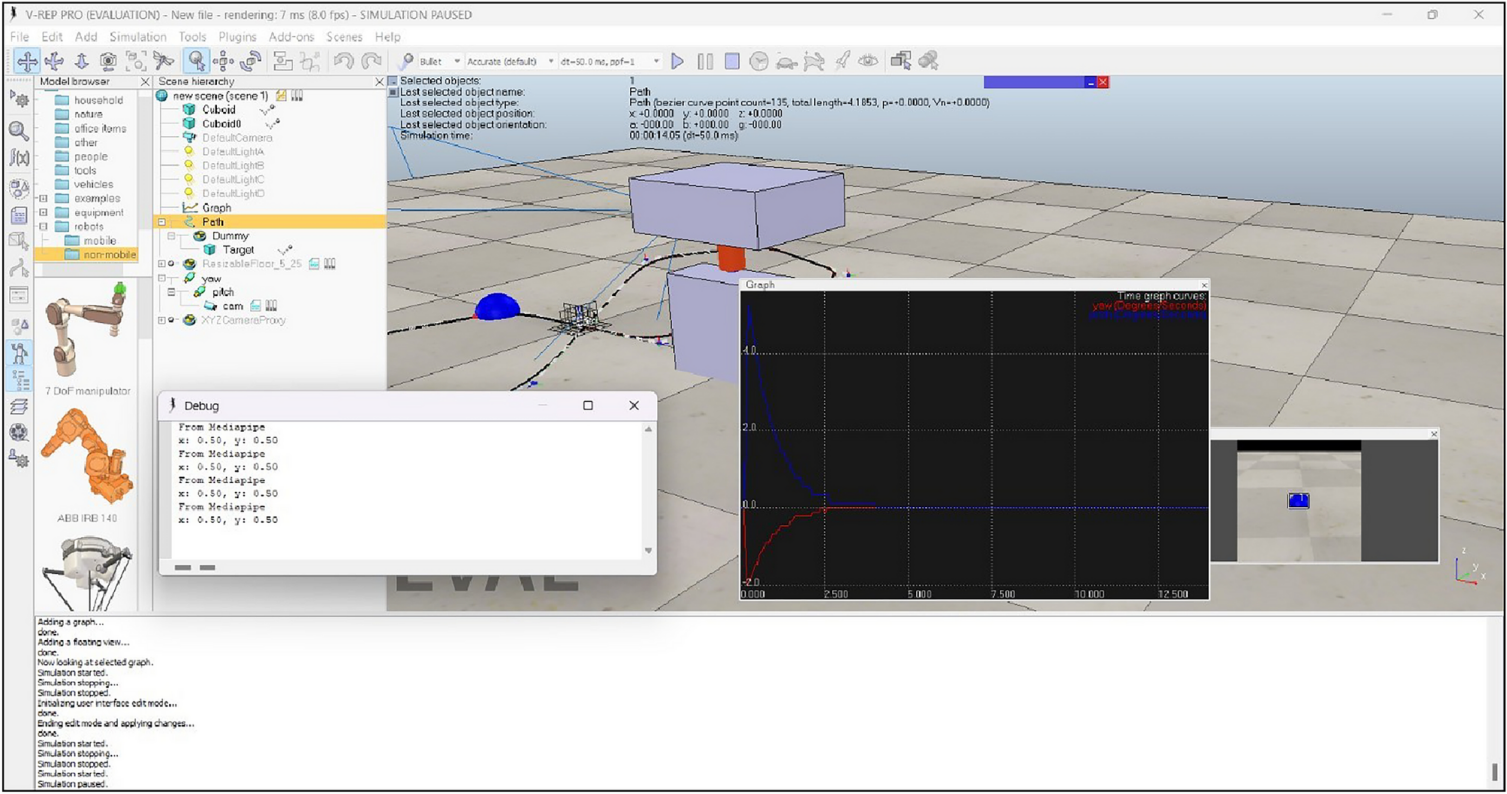
Comprehensive view of the entire coppeliaSim environment.

In the Lua script embedded within the child script of the visual sensor, an IBVS tracking algorithm has been developed to control the joint velocity based on the received *x* and *y* coordinate values. This algorithm ensures continuous tracking of the object. The effectiveness of the tracking algorithm is demonstrated in Fig. 9, where a plot illustrates the variation of joint velocity for both yaw and pitch joints over time. As the object is detected and tracked, the joint velocities adjust accordingly, ultimately reaching zero velocity once the object is stationary. This behavior confirms the successful tracking of the moving object, as reflected by the diminishing joint velocities. Finally, Fig. 10 provides a comprehensive view of the entire CoppeliaSim environment. In the bottom left corner, continuous publication of *x* and *y* coordinate values is evident shown in Fig. 10, accompanied by the concurrent display of the graph and visual sensor output. These visualizations collectively affirm the successful implementation of our tracking algorithm in the simulated environment, reinforcing the robustness and efficacy of our approach.

### Experimental results

**Fig. 11:**
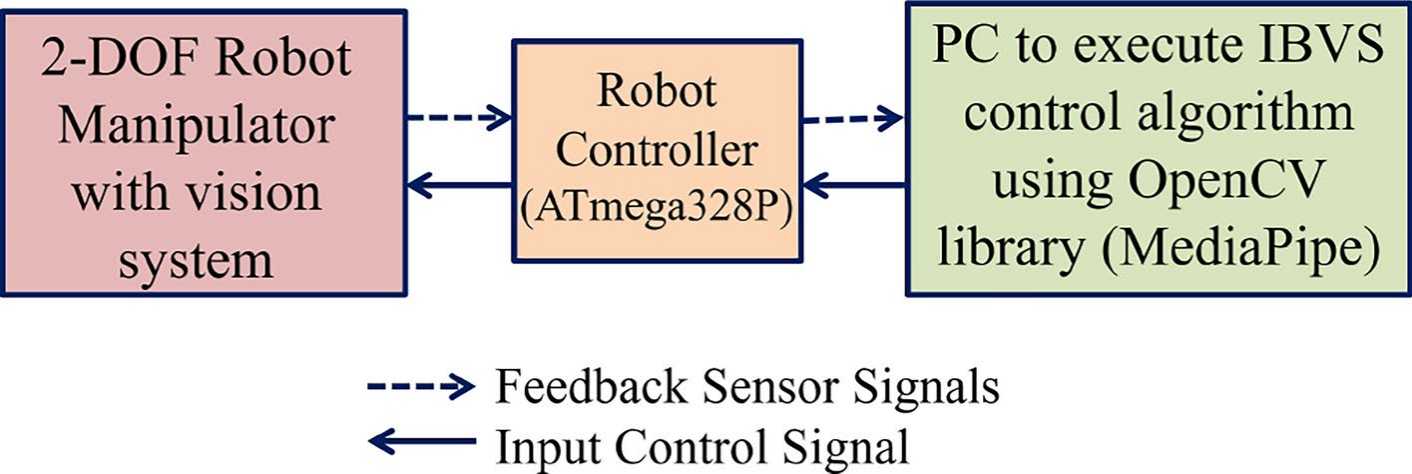
Signal flow of experimental setup.

**Fig. 12:**
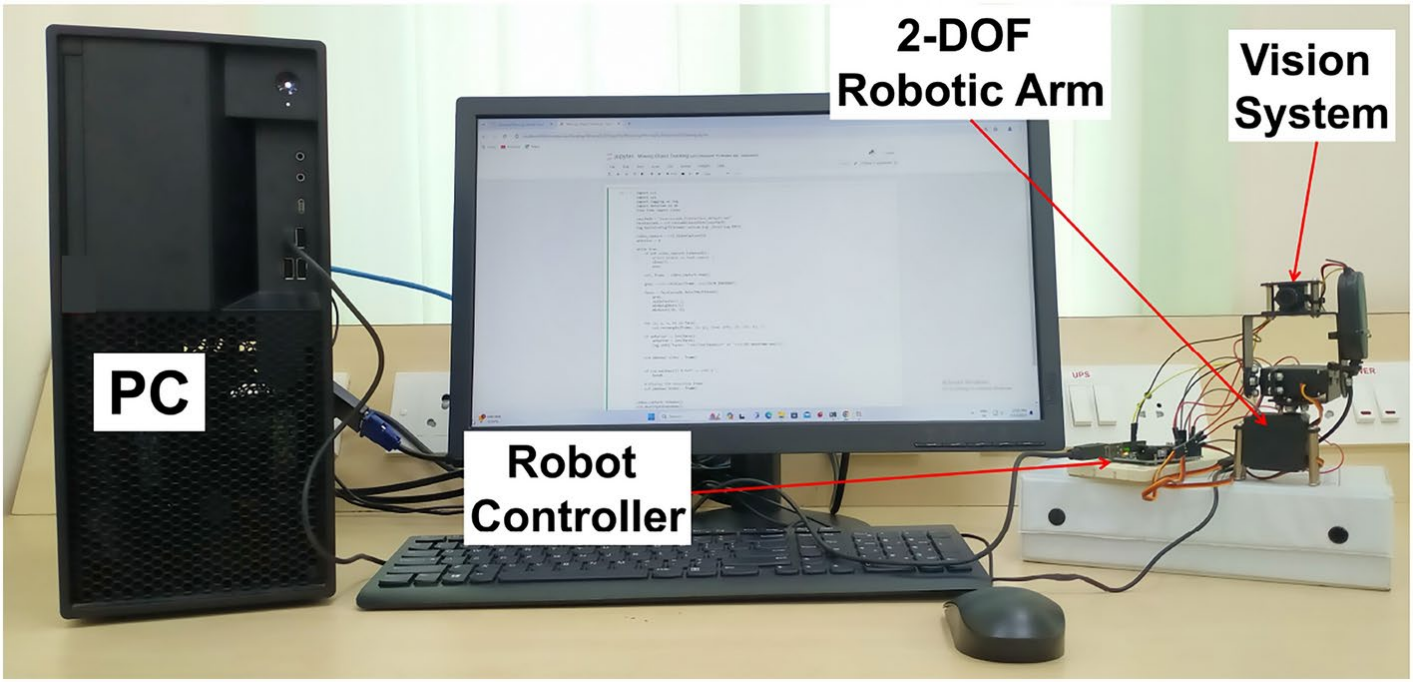
Experimental setup.

**Fig. 13:**
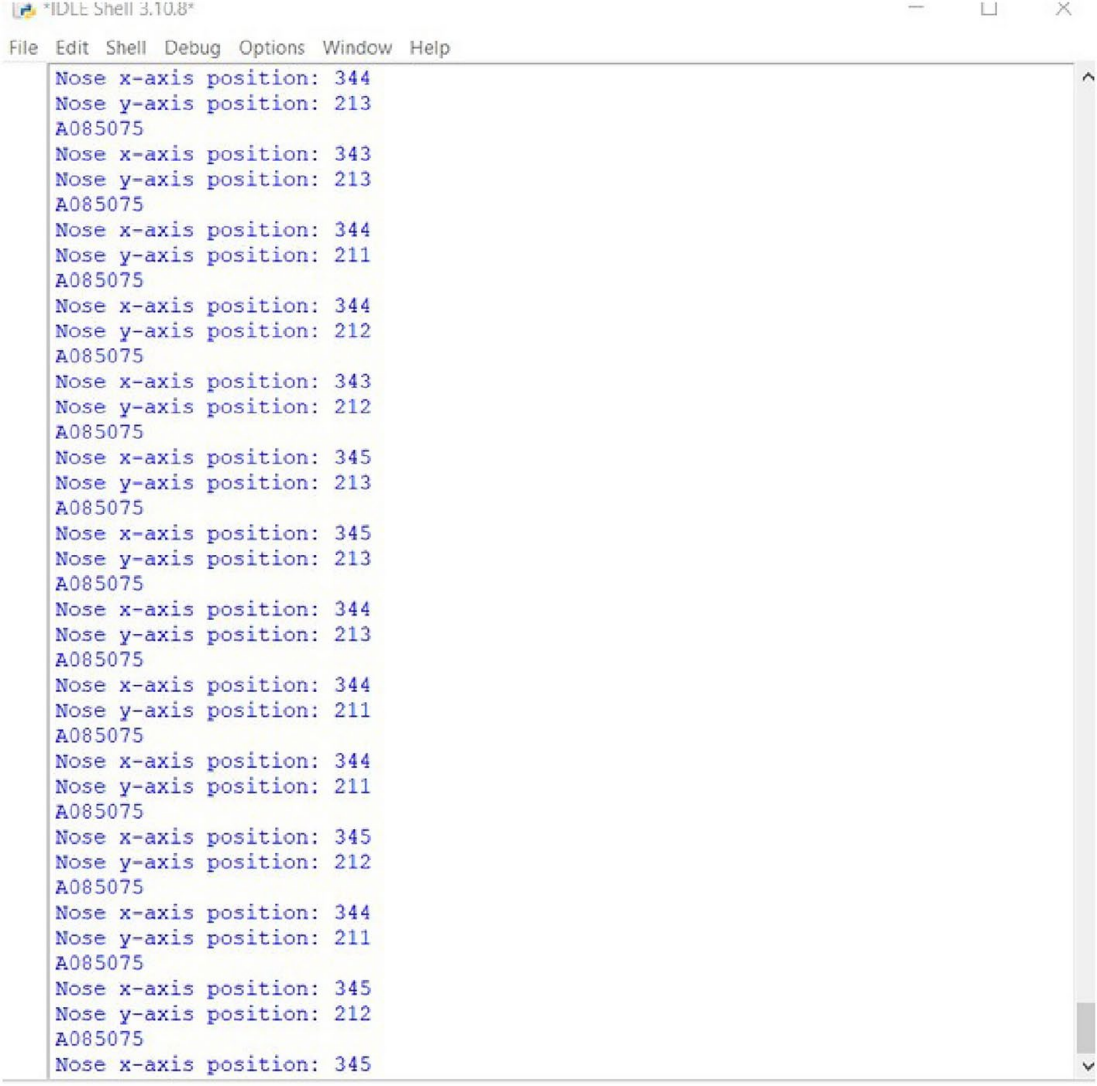
Serial port publishing position of the moving object.

The 2-DOF robotic arm for tracking moving object has achieved a significant milestone in seamlessly integrating a 2-DOF robotic arm with an ATmega328P based Controller, effectively transforming it into a dynamic system capable of tracking a moving object. In this case, the object of interest is a person’s nose, detected through a camera affixed to the robotic arm’s frame. Operating initially in an idle state, the robotic arm autonomously sprang into action upon the detection of the nose, accurately mirroring its movements. The integration leveraged the Mediapipe framework^42^ to extract real-time nose tip coordinates from a video stream. Calibration of the camera enabled the translation of 2D coordinates into their 3D counterparts, ensuring precise tracking in the physical world. A custom algorithm, tailored for object detection and localization, continuously monitored the object’s position and issued precise commands to the controller. This orchestration facilitated synchronized movements of the robotic arm in tandem with the person’s nose. The successful real-time tracking of the nose tip, coupled with the validation of results in real-world scenarios, underscores the robustness and versatility of the implemented IBVS based tracking control system. This accomplishment holds promising implications for applications requiring dynamic object tracking within the realm of robotics. Figure 11 shows the signal flow diagram of experimental setup. Figure 12 shows the experimental setup. In Fig. 12, personal computer (PC) with IBVS control algorithm composed of OpenCV library and MediaPipe framework facilitate integration of software tool with hardware components (2-DOF robotic arm) to achieve visual servoing task. Figure 13 shows the real-time output of nose position w.r.t *x* and *y* coordinates obtained by feature based object detection algorithm and IBVS tracking controller to control the 2-DOF robotic arm.

The methodology involves a Python based IBVS algorithm composed of OpenCV libraries and MediaPipe framework to extract the moving object position in a live video feed from a camera. Once the moving object coordinates are detected, they are translated into specific ‘z’ values based on predefined ranges. These ‘z’ values are then sent to an ATmega328P based microcontroller via serial communication, establishing a direct interface between the Python and Arduino codes. In the Arduino code, the received ‘z’ values are parsed and utilized to control 2-DOF robotic arm. This communication link allows the controller to interpret the moving object position data from Python based IBVS control algorithm and translate it into corresponding movements of the joints, resulting in a responsive and interactive system where moving object directly influence physical actions.

### Discussion

The simulation and experimental results provide clear and compelling evidence that the object tracking system is effective. The outcome demonstrates that the robotic arm successfully tracks a continuously moving object. This is evident from the plot shown in Fig. 9, which shows that the joint velocities of the robotic arm come to zero once the object is tracked, indicating that the arm has reached the desired position and is no longer adjusting its movement. Additionally, Fig. 10 presents the camera view in bottom right corner, clearly showing the object centered in the field of view, confirming that the object is being accurately tracked.

Figures 8, 9, 10, 11, 12 and 13 further support this by illustrating that the object coordinates are continuously tracked and published. These figures show that the coordinate values remain consistent when the object is at rest and tracking is complete, which aligns with the plot shown in Fig. 9 where the joint angles are zero once the object tracking is complete. This consistent results across simulation and experimental results validates the conclusion that the object tracking system is effective. The robotic arm’s ability to bring joint velocities to zero and maintain the object in the center of the field of view demonstrates precise and reliable tracking performance.

### Discussion on data-driven learning techniques

In the literature, recent advancements in data-driven learning have significantly impacted the field of robust control, particularly in adaptive cruise control and nonlinear system regulation. In^46^, authors explored adaptive robust control using data-driven learning for systems with significant uncertainties. In this research work, authors demonstrated about data-driven learning in mitigation of modeling errors and disturbances by offering robust performance in sensor-driven systems. Extending the available techniques, authors developed an adaptive Q-learning-based ${\text{H}}_{\infty }$ control method in^47^ for continuous-time nonlinear systems, which leverages reinforcement learning (RL) to manage non-linearities and enhance system adaptability without prior knowledge of system dynamics. Further in^48^, authors proposed a model-free ${\text{H}}_\infty$ prescribed performance control strategy using policy learning, which eliminates the need for explicit system models and optimizes real-time control adaptability, thus handling system disturbances more efficiently. Additionally, in^49^, authors introduced a framework for data-driven ${\text{H}}_\infty$ control of adaptive cruise control systems, emphasizing robustness and stability while managing uncertainties and external disturbances. The discussed approaches are especially relevant in dynamic vehicular environments where traditional model-based techniques may suffer with some disadvantages. Collectively, the elaborated studies illustrate the potential of integrating data-driven learning techniques such as Q-learning, policy learning, and model-free control into robotic and control systems, laying a foundation for improving adaptability and robustness in dynamic and uncertain environments.

Recent advancements in techniques such as RL and model-free control offer significant opportunities to optimize the performance of our 2-DOF robotic arm in dynamic and unpredictable environments. By integrating these methods with our current IBVS scheme, the robotic arm could gain the ability to adapt more effectively to environmental changes, such as sudden object occlusions or visibility issue or varying lighting conditions, which traditional IBVS may struggle to manage. RL, in particular, could be employed to enable the system to learn optimal control actions through trial-and-error interactions with the environment, improving both robustness and adaptability^17^. Model-free control, which eliminates the need for a detailed dynamic model, would further enhance the system’s flexibility in scenarios with partial or uncertain state information.

Building on these advancements, we recognize the potential of hybrid approaches that combine traditional IBVS with data-driven techniques, such as neural networks, to address challenges like visibility issue, disturbance rejection and noise handling. The present work is capable to handle image noise and moderate external disturbance. However, the proposed control scheme may fail if object is not present in the field-of-view (FoV). Many data-driven learning based approach with VS has been proposed to deal with complex manipulation task such as target tracking^50^, object manipulation^51^, controlling mobile robot^19,52,53^, robot manipulator^54–56^, 2-DOF Helicopter System^57–59^ and servo mechanism^60^. Also, RL^17,61^ and Deep RL (DRL)^62–64^ have been applied in robotic manipulation task. These brief literature revels that neural networks are particularly well-suited for learning and compensating for non-linearities and uncertainties that may not be fully captured by conventional IBVS algorithms, especially in complex, dynamic environments. By embedding such learning mechanisms into the control loop, the system could dynamically adjust the position to keep the object within FoV, adapt to environmental disturbances and sensor noise, thereby improving overall stability and precision.

The vision-based tracking control scheme presented in section “Proposed vision-based tracking control scheme” does not guarantee the retention of visual features within the camera’s FoV. Additionally, higher controller gains lead to increased input torque, causing visual features to move out of the FoV more rapidly, resulting in system instability and reduced accuracy . To address this visibility issue, a hybrid control scheme can be implemented. The proposed hybrid control scheme will incorporate a RL controller, which can be integrated into the existing control framework to address visibility issues. In the proposed hybrid control scheme, the RL controller will adjusts the arm’s position to bring the object within the FoV by selecting the optimal control input, after which the existing control scheme completes the visual servoing task. This hybrid control architecture would enable the robotic arm to anticipate and correct for perturbations that could degrade visual tracking or control accuracy.

### Comparison analysis

In this work, the important difference between the proposed control scheme as compared to other schemes^29–32,35,37,39^ is presented as follows. First, the present work utilizes a DL-based approach for efficient visual feature extraction to address the limitation of IBVS unlike^29–32,35,39^. However, in^37^, a DRL controller is used to learn the control policy for a robotic arm. Second, the integration of machine vision and automatic control technology, combined with a novel dual-coordinate approach utilizing both 2D and 3D spatial information, enhances object detection and localization, effectively addressing the limitations of external perception in handling sudden situations faced by other control methods. In conclusion, the proposed vision-based tracking control for 2-DOF robotic arms offers advantages such as improved control and efficiency, real cooperation of two arms, and integration with machine vision similar to^29–32,35,37,39^.

### Safety aspects of proposed scheme

The proposed control scheme ensures safety through multiple robust mechanisms. Firstly, the implementation of a powerful DL framework like MediaPipe for feature extraction enhances a high level of accuracy and reliability in object detection and tracking. MediaPipe’s pre-trained models, trained on diverse datasets, can recognize intricate patterns and extract point features that remain robust across varying conditions, including changes in lighting, background clutter, and occlusions. The DL models in MediaPipe are trained on extensive datasets, which include a wide variety of conditions and environments, enhancing their ability to generalize and perform reliably in real-world applications. This robustness ensures about consistency and accuracy of a system such that it can be easily identifies the target feature by significantly reducing the risk of detection errors.

Further, the control logic in this approach is designed to be both efficient and reliable. It relies solely on the coordinates extracted from MediaPipe to assign joint angles to the robotic arm. This simplified approach minimizes the complexity of the control system by reducing the likelihood of software bugs or malfunctions. The utility of 2D coordinates to control the joint angles and the 3D coordinates to regulate the speed confirms that the system achieves precise and responsive movements. This dual-coordinate approach enhances the accuracy of the robotic arm’s actions, ensuring that it can safely and effectively track the moving object.

Moreover, real-time feedback integration within the control loop allows continuous monitoring and adjustment based on the object’s position. This dynamic adaptability enables the robotic arm to respond swiftly to environmental changes, ensuring stable and reliable operation. Overall, the combination of advanced DL techniques, robust feature extraction, and a simple yet effective control logic contributes to the safety and reliability of the proposed system.

## Conclusion and future scope

The presented study proposes IBVS-based innovative tracking control of a 2-DOF robotic arm to overcome the challenges related to sensing and stability concerns in real-time object tracking with robot manipulator. The proposed control scheme integrates a precise deep learning based moving object detection algorithm with an efficient IBVS tracking controller to achieve the visual servoing task. The feature-based object detection algorithm is utilizes OpenCV libraries to process the input image data. Moreover, the MediaPipe framework incorporated a deep learning approach to detect the visual feature from moving objects by ensuring robust and adaptive tracking performance. Further, the selected deep learning based features address the issues associated with the IBVS approach. This proposed scheme was thoroughly investigated through simulations and real-time experiments, yielding expected results with the successful tracking of the moving object. The developed IBVS tracking control algorithm, driven by error feedback in both directional coordinates, demonstrated the system’s ability to continuously track the object with remarkable precision.

In the future, enhancing the proposed control scheme with data-driven learning techniques can improve the robustness and adaptability of object tracking. The proposed control scheme can also be applied to perform various tasks under different conditions to cater to industrial and medical applications.

## Data Availability

The data used and/or analyzed during the current study are available from the corresponding author upon reasonable request.
