# Publisher Correction: Autonomous object tracking with vision based control using a 2DOF robotic arm

**DOI:** 10.1038/s41598-025-02633-4

**Published:** 2025-05-21

**Authors:** Umesh Kumar Sahu, Mebin K. S., Abhinav K., Muhammed Muzammil P, Ankur Jaiswal, Umesh Kumar Yadav, Santanu Kumar Dash

**Affiliations:** 1 Department of Mechatronics, Manipal Institute of Technology, Manipal Academy of Higher Education, Manipal, Karnataka 576104 India; 2 Department of Electrical Engineering, Malaviya National Institute of Technology, Jaipur, Rajasthan 302017 India; 3 TIFAC-CORE, Vellore Institute of Technology, Vellore, 632014 India

Correction to: *Scientific Reports* 10.1038/s41598-025-97930-3, published online 18 April 2025

In the original version of this Article, Santanu Kumar Dash was omitted as a corresponding author. Correspondence and requests for materials should also be addressed to: santanu4129@gmail.com.

The original Article has been corrected.

